# The Week's Work

**Published:** 1910-02-26

**Authors:** 


### THE WEEK'S WORK.

### CHESTER GENERAL INFIRMARY.

Sir Henry Burdett's report upon this institution is held over by request for the present. As the Mayor, Mr. Alderman Hewitt, has intimated, there is need for courageous action on the part of the Governors, and a little delay may possibly help forward a movement which should have behind it the best opinion and support of the inhabitants of Chester city and of Cheshire too.

### THE LAST STAGE OF TUBERCULOSIS.

We understand that it is proposed to erect buildings in connection with the Royal Hospital for Incurables in Donnybrook, Dublin, containing 60 to 100 beds, for the reception of tubercular cases in the last stage of the disease. This is the first home for the dying of this type which we have yet heard of. The cost should be relatively small, though it is essential the buildings should be completely isolated. We shall watch the development of this novel proposal with special interest, for it may indirectly help to make the modern sanatoria for tubercular cases in the first stage much more efficient and useful than has heretofore been possible owing to the admission of unsuitable cases from motives of charity.

### THE CHICHESTER INFIRMARY.

The reports of the annual meeting of the Chichester Infirmary which have been published, of which we gave a summary in "Institutional Notes" last week, have led to some misapprehension. The sub-committee appointed by the Governors to consider the financial position made 21 recommendations, of which one was rejected, that relating to the issue of one out-patient ticket for each subscription of 5s. per annum. Two were withdrawn relating to payments by in-patients which had already been put into force, and the other imposed a registration fee of 6d. upon out-patients, of which the medical staff disapproved. Two were referred back; one of these imposed a new scale of privileges for annual subscribers, and we regret to see that the committee's recommendation to raise the subscription for in-patient letters to 3 guineas and upwards was rejected. Three guineas is the smallest subscription for which any in-patient ticket can be issued by a modern hospital where any regard is paid to the cost of in-patient maintenance in the present day. The other recommendation to refer back related to the establishment of a reserve fund of £20,000, and is one that we hope may be ultimately accepted, in principle at least. All the other recommendations were approved. They include the issue of special appeals by the Mayors of Chichester and Arundel and the Chairmen of District Councils; more active co-operation on the part of the Governors in raising funds; the institution of various efforts to raise money, including a special effort by the clergy and ministers of all denominations during 1910; an invitation to the Governors to underwrite a sum of not less than £10 each, which they undertake to give or raise within a fixed period and to pay over to the Treasurer; an earnest endeavour to increase the subscription list and the amount of individual subscriptions, and by direct personal appeal to induce business firms and companies to subscribe and interest themselves in raising funds. The artisans are asked to take boxes and give personal service in the same cause; women and children of all classes to make strenuous efforts to raise penny subscriptions in each of the parishes from which a patient is sent; a thorough organisation of every district from which patients are received, including the establishment of a Ladies' Association or Guild; the appointment of a secretary and superintendent to devote his whole time to the business of the Infirmary. It will be seen that a wide field is to be covered, which is wise, seeing that this infirmary needs an additional income of £1,000 a year. Providing the recommendations of the Committee as adopted are taken up in the right spirit that sum should easily be raised, with the assistance of a paid secretary, before the date of the next annual meeting. The hearty thanks of the Governors are due to the sub-committee, and especially to the Bishop, the Mayor, and to Major Jellicourse, its Chairman.

### THE NEW GENERAL HOSPITAL, TORONTO.

A bird's-eye view of the proposed new buildings of the Toronto General Hospital is being circulated in support of a public appeal for the £200,000 which is yet required to complete the building fund of £500,000. The plan shows that the general wards are placed in front in a series of pavilions of three stories, with an administration block of four stories in the centre. There is a large two-storied out-patient department, and a small emergency or casualty building of one story. There is a large building of four stories for private and semi-private patients, with accommodation for 98 beds; an obstetrical block of two stories; a pathological building; a nurses' home of five stories, with accommodation for 174 nurses; a three-story building for the servants; and a central power house. The new hospital will contain 449 beds for free cases, in addition to the 98 pay beds. The estimated total cost, including equipment, is £500,000. Of this £300,000 is in hand, and of the balance of £200,000, it is expected that £40,000 will be provided by the City Corporation. We shall look forward to the detailed plans and particulars of this hospital with hopeful expectation. The old General Hospital has done a magnificent work, and we hope the new one may worthily carry on the best traditions and efficient administration which have made this institution so full of interest in the past.

### THE HOSPITAL SUNDAY FUND IN IRELAND

There has been a tendency in Ireland during the last few years for the subscriptions to the Hospital Sunday Fund to diminish steadily. It is impossible to say definitely to what this decrease, which this year amounts to over three hundred pounds, is due, and it would serve no good purpose to speculate on all the possible causes. This much, however, seems certain, that there are several factors at work which influence the subscribers in reducing the amount of their subscriptions. The *Dublin Express* has recently devoted some attention to the subject, and concludes that increased taxation of incomes is principally responsible for the reduction, and that the deficit is due to the "decreased giving power of those classes which were formerly the mainstay of our charitable institutions." The various denominations in Ireland, with the exception of the Catholic Church, support the Fund, and it seems a great pity that this large religious community should remain apart, and, while not opposing the Fund, at least not actively, refrain from working for it. The contributions towards the Sunday Fund are spent upon the Dublin hospitals, and these institutions exist for the benefit of the poor. They give, and have given, their bounty in work and kind, in conscientious effort and self-sacrificing personal service on the part of the medical and nursing staff, to the sick poor, irrespective of creed or denomination, and it is certain that by far the largest percentage of the patients who benefit by their bounty are Catholics. An objection urged against the Fund in Dublin is that there are too many hospitals in that city, and that these are already sufficiently well endowed. That is an objection which should not weigh with those who are acquainted with the facts. The Dublin institutions are not better endowed than those in other large cities: they are in frequent financial difficulties, and they can scarcely be said to be adequate for the "hospital population" of the city. The arguments in favour of contributing to the Sunday Fund are cogent, and should have weight with the charitable, and it is sincerely to be hoped that the efforts now being made to promote an active interest in the Fund will result in a progressive increase in the annual total contribution to the Fund.

### "DECOY DUCKS."

Sir Henry Burdett, in several reports already published, and again this week in the case of the Royal Berkshire Hospital, recommends the authorities to develop the private nursing staff and to give to each of their subscribers the privilege of having the first claim to the services of a trained nurse, whose character can be guaranteed, whenever family needs may require such service. The advanced advocates of nurse registration, who never tire of endeavouring to prejudice trained nurses against private staff appointments, have misrepresented Sir Henry's recommendations in an article under the above heading which has appeared in their official organ. This is done by claiming that Sir Henry's recommendation would amount in practice to an attempt on the part of the hospital authorities to exploit a trained nurse for gain, contrary to the nurse's welfare. This is, of course, sheer nonsense. Where the private nursing staff is properly organised and adequately paid, as at Guy's Hospital, there is no field of work open to those who have a gift for private nursing so satisfactory, on the whole, as the private staff of a great hospital. Further, it is good for the nurse, as it is good for the subscribers and authorities of each institution, that the most cordial co-operation should be continuously maintained between all these interests for their mutual well-being. We may add that in the case of no up-to-date, well-administered British hospital are the nurses permitted to canvass for subscriptions or donations from their patients for charitable purposes of any kind. The official organ properly maintains that both patients and nurses would be placed in a false position were such canvassing permitted. But it is amusing to read the protest against well-deserved recognition being given to those permanent officials to whose devotion and service the efficiency of the administration and the comfort of the inmates of a hospital may be mainly due. There is no force of greater value in the management of a great institution than the wisdom which provides that adequate recognition shall be extended to everyone who zealously labours in the cause of the sick.

### PAYMENTS TO PRACTITIONERS WHEN SUMMONED BY MIDWIVES.

The letter of Mr. Adair Dighton which we published last week, page 616, raises an important point. Mr. Dighton asks to whom is he to look for his well-earned fee of two guineas in the case mentioned. The answer is, to the Winchcombe Board of Guardians, to whom he was properly referred by the Medical Officer of Health for Gloucestershire. The case is provided for in a circular issued by the Local Government Board, dated February 18, 1910. In that circular the Board lays it down that they "regard it as of immediate importance that medical practitioners should, so far as practicable, feel assured of a reasonable payment for their services in these cases." To secure this, they "impress upon Boards of Guardians that they should give effect" to the Board's "circular letter of July 29, 1907." This provides "that medical men and certified midwives practising in the Poor Law Union should be informed that, as regards any poor person in whose case the attendance of a registered medical practitioner is required, the Guardians will be prepared to exercise their powers under Section 2 of the Poor Law Amendment Act, 1848, and to pay a reasonable remuneration to the medical man called in." It is suggested that any medical practitioner preferring such a claim should state definitely that after making reasonable efforts he has failed to secure payment from the person attended. When Mr. Adair Dighton has followed the course laid down by the Local Government Board and obtained his fee, or otherwise, we shall be glad to hear from him again.

### THE DRUG MARKET.

The drug market is singularly quiet, and the improvement which was so confidently expected early in the New Year is very slow in making its appearance. Notwithstanding the very slow demand for drugs and chemicals prices are well maintained on the whole, although in a few cases there is a weaker tendency, which may lead to lower prices if orders do not come in. Cod-liver oil is still attracting attention, and the position has not improved since last week. The downward tendency in the price of Buchu leaves continues, but supplies are still very small. As to actual changes in value, there have been few of any importance during the week.

